# Vaporization of Intact Neutral Biomolecules Using Laser-Based Thermal Desorption

**DOI:** 10.1021/jasms.3c00194

**Published:** 2023-06-15

**Authors:** Yerbolat Dauletyarov, Siwen Wang, Daniel A. Horke

**Affiliations:** † Institute for Molecules and Materials, Radboud University, Heyendaalseweg 135, 6525 AJ Nijmegen, The Netherlands

## Abstract

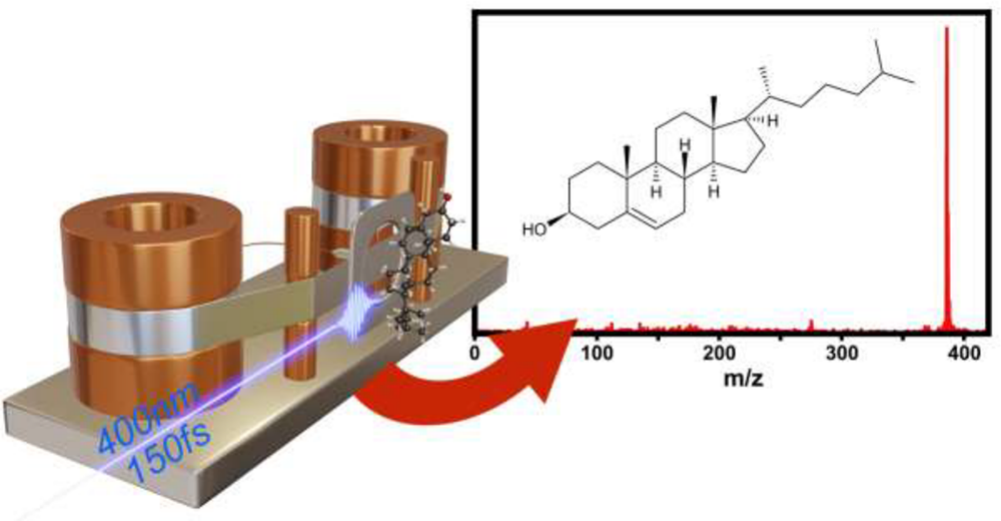

The production of a clean neutral molecular sample is a crucial step in many gas-phase spectroscopy and reaction dynamics experiments investigating neutral species. Unfortunately, conventional methods based on heating cannot be used with most nonvolatile biomolecules due to their thermal instability. In this paper, we demonstrate the application of laser-based thermal desorption (LBTD) to produce neutral molecular plumes of biomolecules such as dipeptides and lipids. Specifically, we report mass spectra of glycylglycine, glycyl-l-alanine, and cholesterol obtained using LBTD vaporization, followed by soft femtosecond multiphoton ionization (fs-MPI) at 400 nm. For all molecules, the signal from the intact precursor ion was observed, highlighting the softness and applicability of the LBTD and fs-MPI approach. In more detail, cholesterol underwent hardly any fragmentation. Both dipeptides fragmented significantly, although mostly through only a single channel, which we attribute to the fs-MPI process.

## Introduction

Delivery of biomolecules, large and small alike, into the gas-phase is the main prerequisite for successful application of gas-phase spectroscopy and reaction dynamics techniques to molecular biology. Although the invention of electrospray ionization (ESI) and matrix-assisted laser desorption and ionization (MALDI) was a huge leap forward for ionic species, the progress for neutral species was fairly modest in comparison. The main problem with neutral species is *non*-applicability of ion-optics-based mass-filtering to isolate a molecule/cluster of interest from neutral contaminants resulting from fragmentation and/or clustering. This is especially relevant when an experiment involves so-called “universal probes” such as femtosecond (strong-field) ionization or X-ray/electron scattering, as opposed to selective methods such as resonance-enhanced multiphoton ionization.

Historically, neutral molecular beams of compounds of interest were produced by supersonic expansion of their vapors seeded, by heating, in a carrier gas. By its very nature, this method was limited to volatile and thermally stable compounds, precluding the study of large and many small biomolecules. A major advance here was the development of laser desorption jet-cooling (LDJC)^1^ introduced in the late 1980s. In this method, a neutral molecular beam is produced through desorption of a target compound, placed on or mixed with a suitable surface material (typically graphite) by an IR laser pulse directly in front of an expanding supersonic jet of a carrier gas. Since then LDJC has been very successfully combined with various spectroscopic techniques to study a range of biomolecules including nucleobases,^2^ nucleosides,^3^ peptides,^4^ and peptide aggregates.^5^ However, it has been shown that it may cause considerable fragmentation of a target molecule, contaminating a molecular beam.^6^ In addition, the surface material itself is a potential source of contamination. Furthermore, typical repetition rates for LDJC are on the order of 10 Hz, which considerably limits the experimental throughput.

More recently, Greenwood and co-workers^7^ introduced an alternative approach, namely laser-based thermal desorption (LBTD), in their study of proton-impact fragmentation of neutral DNA nucleosides, known to be thermally labile. It is based on an older technique, laser-induced acoustic desorption (LIAD),^8^ which has been used to desorb neutral nucleobases, dinucleoside phosphates, amino acids, lipids, and small peptides.^9−13^ In both approaches, a sample is deposited on a substrate (typically a foil made of metal with a high melting point) and desorbed into the gas-phase by irradiating the back side of the substrate with a laser, a continuous one in LBTD and a pulsed one (typically with nanosecond pulsewidth) in LIAD. One of the advantages of LBTD/LIAD over LDJC is the absence of any additional material such as graphite, since the sample is typically deposited by drying its solution on a metal substrate. While technologically quite similar, the desorption mechanisms for LIAD and LBTD differ significantly. While LBTD is clearly a form of soft thermal vaporization,^14,15^ the mechanism for LIAD, initially thought to be purely acoustic, is still actively debated.^16,17^

The LBTD approach has now been implemented by a number of groups around the world, and has successfully vaporized nucleobases,^14,18^ amino acids,^19−21^ and nucleosides.^7,22^ Related approaches that use a glass surface instead of a metal substrate have also been demonstrated for desorption of nanoparticles^23^ or phthalocyanine molecules.^24^

A major advantage of the LBTD approach is that the use of a continuous desorption source makes this an inherently continuous molecular source, in contrast with the pulsed LIAD approach. This allows its combination with high repetition-rate ionization lasers, as we demonstrate here, for high-throughput data collection. The availability of cheap continuous diode lasers furthermore makes LBTD much more cost-effective.

In this contribution, we demonstrate that the LBTD approach is also applicable for the vaporization of fragile and nonvolatile biomolecules. In particular, we report mass spectra of two dipeptides (glycylglycine and glycyl-l-alanine) and a lipid (cholesterol) obtained using LBTD combined with femtosecond multiphoton ionization (fs-MPI) at 400 nm. Notably, because of its thermal instability, the structure of glycylglycine was not known until very recently, when it was determined for the first time using rotational spectroscopy.^25^ In all cases, the obtained mass spectra contained a significant contribution from intact precursor ions, significantly more than available reference spectra obtained using electron impact ionization. The remaining fragmentation of the dipeptide samples is primarily attributed to the fs-MPI process.

## Experimental Methods

**Figure 1:**
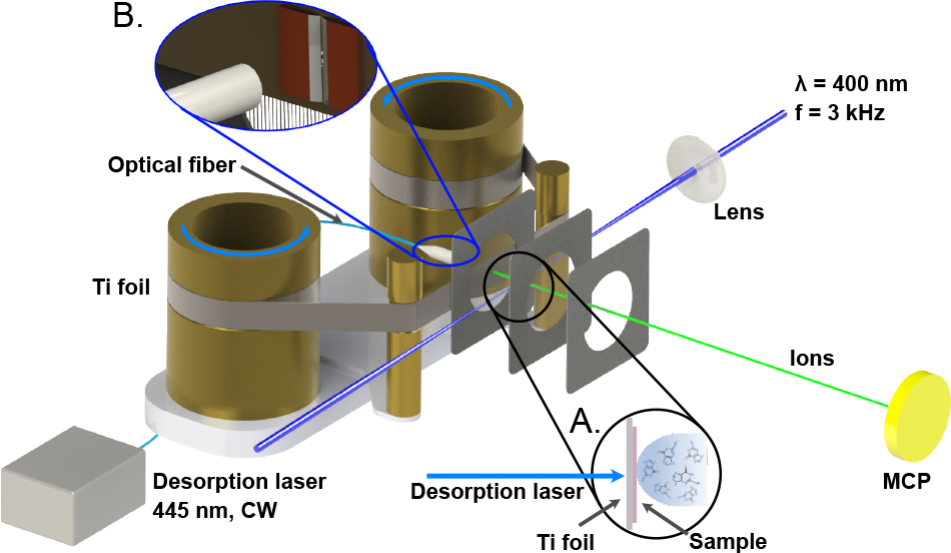
Schematic of the laser-based thermal desorption source. The sample is deposited onto a thin titanium foil, which is held by two rotating rollers constantly providing a fresh sample. A continuous desorption laser irradiated the back of the foil to vaporize samples. Molecules are ionized by femtosecond multiphoton ionization, and ions are detected in a custom time-of-flight mass spectrometer operated in ion counting mode. See the text for further details.

A detailed description of our LBTD-coupled mass spectrometer was given previously,^15,17^ and we detail here only the parameters pertinent to the current study. A schematic of the setup is shown in Figure 1. Sample molecules were deposited onto a 10 μm-thick titanium foil (Baoji Energy Titanium Co.) by spraying their solutions using a commercially available airbrush gun (Fengda, BD-208, 0.2 mm nozzle, ∼2 bar N_2_), and subsequently drying under air. Glycylglycine (Sigma-Aldrich G1002, 99%) was sprayed from its water/ethanol (50%/50% by volume) solution (80 mM) and glycyl-l-alanine (Sigma-Aldrich 50150, 99%) from its aqueous solution (15 mM). Cholesterol (Sigma-Aldrich C8667, 99%) was sprayed from its pure ethanol solution (3.0 mM). Deposited molecules were desorbed into the gas-phase by irradiating the uncoated side of the moving titanium foil (50 μm/s) with the continuous output of a diode laser (445 nm, Wavespectrum Laser Inc.) as shown in inset A in Figure 1. Two knife edges were used to reduce the effective irradiation area on the foil to an approximate size of 3 × 0.2 mm^2^ (see inset B in Figure 1). The diode laser power onto the foil (i.e., measured after transmission through the fiber and the knife edges) was set to 0.1 W for glycylglycine, 0.24 W for glycylalanine, and 0.05 W for cholesterol.

The plume of desorbed molecules was ionized by the second harmonic (400 nm) of a Ti:sapphire femtosecond laser system (Spitfire Ace, Spectra Physics) with a fundamental output at 800 nm, operated at a 3 kHz repetition rate. Typical pulse durations were 150 fs (fwhm). The ionization laser was focused into the interaction region using a plano-convex spherical lens with a focal length of 500 mm, yielding a typical spot size of ∼50 μm in diameter. Laser polarization was kept linear and parallel to the extraction electrodes. Glycylglycine and cholesterol were ionized by 4.5 and 5 μJ pulses, respectively. Mass spectra of glycyl-l-alanine were obtained at three different pulse energies: 6, 10, and 22 μJ.

Produced ions were analyzed using a custom-built Wiley–McLaren time-of-flight mass spectrometer, operating in ion counting mode and with a typical mass resolution . Single ion hits on the MCP detector were recorded and time-stamped by using a combination of constant fraction discriminator (Surface Concept GmbH) and time-to-digital converter (cronologic GmbH).

## Results and Discussion

**Figure 2:**
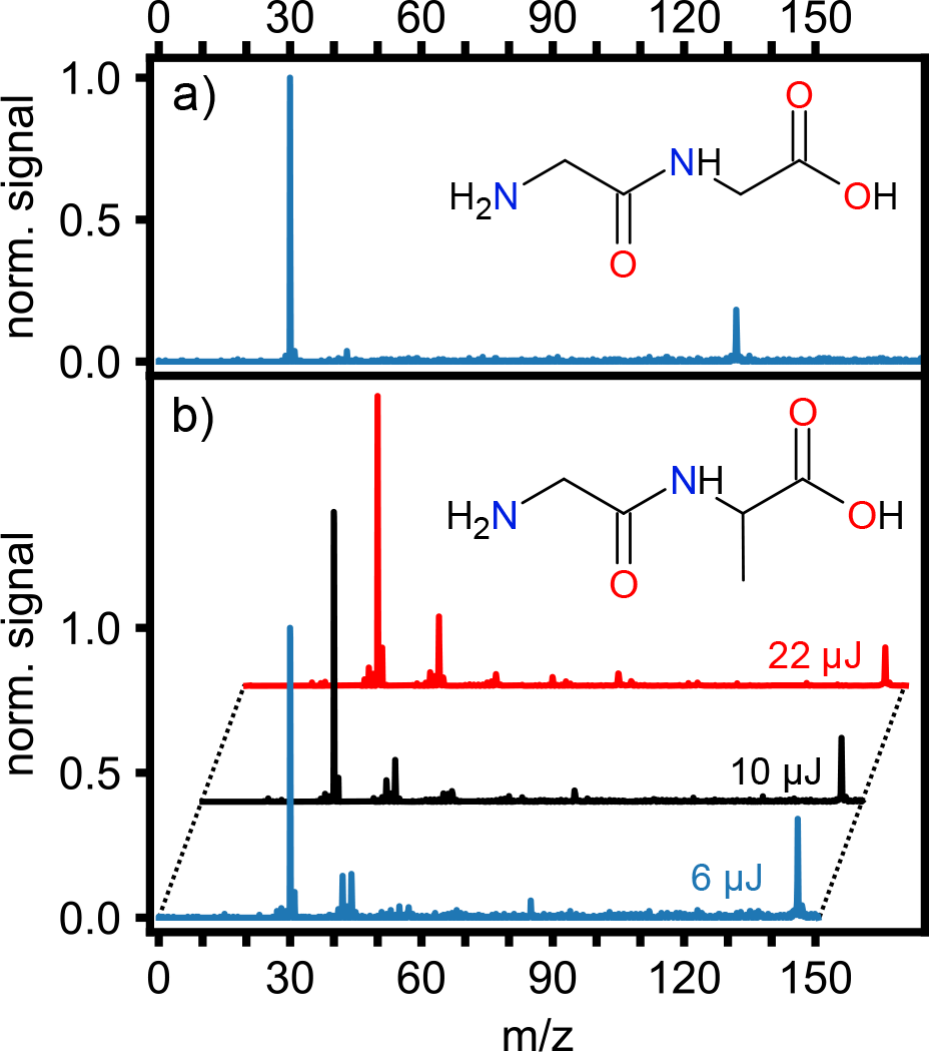
Molecular structures and normalized mass spectra of (a) glycylglycine and (b) glycyl-l-alanine obtained by using LBTD and fs-MPI at 400 nm. Glycylglycine was ionized by 4.5 μJ laser pulses. Mass spectra of glycyl-l-alanine were obtained at three different ionization laser pulse energies: 6, 10, and 22 μJ, corresponding to blue, black, and red traces, respectively. Spectra are normalized to the most abundant fragment and offset for clarity.

In Figure 2, we show normalized mass spectra of (a) glycylglycine (gly-gly) and (b) glycyl-l-alanine (gly-ala) obtained by combining LBTD and fs-MPI at 400 nm, along with their molecular structures. For both molecules we clearly observe a strong precursor ion peak. The mass spectrum of gly-gly, ionized using 4.5 uJ pulses at 400 nm, is very clean, consisting of two major peaks: the precursor ion peak at *m*/*z* = 132 and the most intense fragment ion peak at *m*/*z* = 30. We assign the *m*/*z* = 30 peak to the immonium ion  (*a*_1_), corresponding to the amine end of gly-gly. After losing , gly-gly undergoes, with a low probability, further fragmentation producing CONH^+^ fragment ions corresponding to a small peak at *m*/*z* = 43. To the best of our knowledge, no fragmentation mass spectra of neutral gly-gly have been reported to date, except the one provided in the NIST Chemistry Webbook,^26^ which shows a rich fragmentation pattern typical for electron-impact ionization mass spectra. Nonetheless, *m*/*z* = 30 was the most abundant ionic fragment peak present in the spectrum. The immonium ion *m*/*z* = 30 was also the main ionic fragment in collision-induced dissociation of protonated gly-gly in energy ranges from 3.5 to 7 eV,^27^ and this is furthermore also consistent with computational studies of the fragmentation of protonated glycine.^28^ In contrast, in collision-induced dissociation of protonated cyclic-glycylglycine it was significantly less abundant than other ionic fragment peaks.^29^

The mass spectra for gly-ala are shown in Figure 2b for three different ionization laser energies (and hence intensities): 6, 10, and 22 μJ (blue, black, and red traces respectively). At the lowest intensity, the obtained spectrum is very similar to that of gly-gly, dominated by a single fragment channel of *m*/*z* = 30, which we again assign to the *a*_1_ fragment , and still showing significant signal from intact precursor ions. Increasing the ionization laser intensity decreases the relative intensity of the intact precursor ion while leading to additional fragmentation. In particular, this leads to the opening of a second major fragmentation product at *m*/*z* = 44, corresponding to the immonium ion of *y*_2_*a*_2_ cleavage.^30,31^ This increase in observed fragment yield as the ionization laser intensity is increased clearly indicates that, as in previous studies, the observed fragmentation is primarily due to the fs-MPI process, and not the LBTD source.^15^ The observed fragmentation is again consistent with the available NIST reference spectrum following electron-impact ionization.^26^

**Figure 3:**
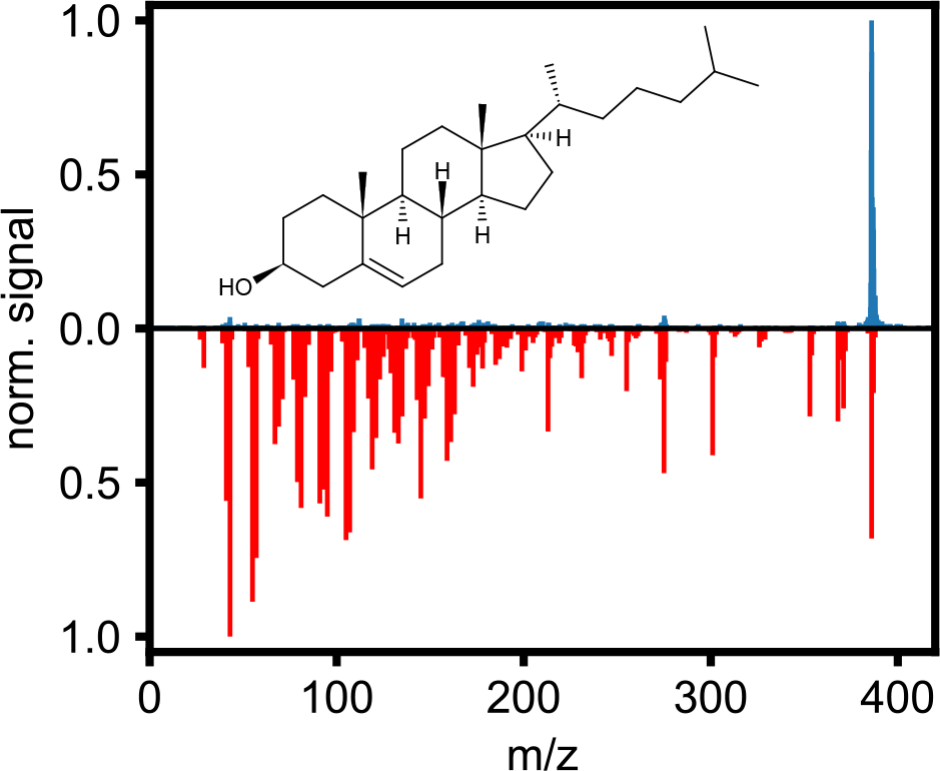
Normalized mass spectrum of cholesterol (blue) obtained using LBTD and fs-MPI at 400 nm. For comparison, the normalized mass spectrum of cholesterol from the NIST Chemistry Webbook,^26^ obtained by means of electron-impact ionization, is shown in red.

In Figure 3, we show the molecular structure and normalized mass spectrum (blue) of cholesterol that we obtained using LBTD and fs-MPI at 400 nm. For comparison, we also show the normalized mass spectrum (red) of cholesterol from the NIST Chemistry Webbook,^26^ obtained by means of electron-impact ionization. Whereas the NIST spectrum shows a very rich fragmentation pattern typical for electron-impact ionization of cholesterol,^32^ fs-MPI combined with LBTD produced a very clean mass spectrum containing a very intense precursor ion peak with almost no fragmentation. Nevertheless, our mass spectrum does contain some of the bands present in the NIST spectrum, albeit in a very small amount, with the most prominent ones being at *m*/*z* = 43, 275, 368, and 371. Cholesterol has also been previously studied using LIAD desorption, coupled to a VUV atmospheric pressure photoionization source.^33^ While these studies also observed a significant contribution from intact precursor ions, the mass spectra were dominated by the  peak. Nonetheless, they showed significantly reduced fragmentation compared to previous VUV-desorption ionization experiments.^34^ This again highlights the softness of our LBTD/fs-MPI approach, and the clear difference between LIAD and LBTD; while the mechanism for LIAD is complicated and still not well understood,^16,17^ LBTD is a thermal process relying on controlled and limited heating of the metal substrate.^14,15^

## Conclusion

We demonstrated that LBTD in combination with fs-MPI can be very effectively used to produce clean, intact, and continuous molecular samples of thermally labile and nonvolatile neutral biomolecules, such as (di)peptides and lipids. Our results show that the vaporization process is extremely soft, with most of the observed fragmentation attributed to the ionization rather than the desorption process.

The presented methodology for producing intact neutral biomolecules in the gas-phase will open up plenty of new opportunities to study biomolecular processes and interactions using the advanced tools of gas-phase spectroscopy and reaction dynamics. Since the produced samples are clean and free from contamination, they also allow the use of “universal probes”, such as ultrafast lasers for time-resolved dynamics studies or X-ray/electron beams for scattering experiments.^35^ The continuous nature of this molecular source furthermore allows for very high-throughput experiments if high repetition-rate lasers are used.^18^ Finally, decoupling the vaporization step from the ionization step allows for greater control and flexibility over the latter.
